# Product development and characterization of a lipid-based *Ayurvedic* polyherbal formulation: *Kalyanaka Ghrita*

**DOI:** 10.1016/j.jaim.2024.101011

**Published:** 2024-10-15

**Authors:** Yashika Singh, Amzad Ali Ansari, Rajendra Prasad Sharma, Saroj Moreshwar Parhate, Thakur Rakesh Singh

**Affiliations:** a Department of Rasa Shastra and Bhaishajya Kalpana, National Institute of Ayurveda, Deemed to be University (*De-Novo*), Jaipur, Rajasthan, India; b Ayurvedic Medical Officer, State Ayurvedic Hospital, Banki Khurd, Ghazipur, Uttar Pradesh, India; c Department of Rasa Shastra and Bhaishajya Kalpana, Shri Narayan Prasad Awasthi Government Ayurved College, Raipur, Chhattisgarh, India

**Keywords:** *Kalyanaka Ghrita*, *Sneha Kalpana*, Standardization, HPLC, GC-MS

## Abstract

**Background:**

*Kalyanaka Ghrita* (KG) is polyherbal oleaginous medicament consisting of extracts from twenty-eight different plants, indicated for management of psychosomatic disorders like *Unmada* (Schizophrenia), *Apasmara* (Epilepsy) and numerous other ailments.

**Objective:**

To develop and validate standard manufacturing procedure of KG by following *Ayurvedic* principles in three batches to ensure process uniformity and standards.

**Materials and methods:**

Three batches of KG were prepared by adopting principles of *Ashtanga Hrudya* and Ayurvedic Formulary of India to ensure consistency in manufacturing process. Observations during process such as temperature, duration were recorded. KG was subjected to chief desired characteristics, organoleptic (color, odour, taste, texture, touch), physicochemical (acid value, peroxide value, iodine value, saponification value, loss on drying, refractive index, specific gravity, mineral oil, rancidity test, viscosity) as per pharmacopeial standard. Chromatographic screening and fingerprinting of KG were conducted through GCMS whereas quantification of curcumin and chebulagic acid biomarkers were assessed through HPLC.

**Results:**

Average yield of KG was 83.41%, with average intermittent heating duration of 20.35 h subsequently divided into three days. Temperature throughout preparation ranged from 66 °C to 101 °C. KG was pale olive in colour, exhibiting pleasant taste, characteristic smell, and soft texture. Organoleptic and physicochemical characters were comparable for three batches of KG while safety parameters were found within permissible limits.

**Conclusion:**

Pharmaceutical standardization of *Kalyanaka Ghrita* is necessary for establishing biological and chemical profile of formulations. Present study recommends use of coarse powdered ingredients for optimal yield during pharmaceutical process, and heating up to *Madhyama Paka* stage calibrated over three days with average temperature of 85 °C. The data obtained from this study may contribute to future research and development activities, serving as a basis for manufacturing standards of KG.

## 1 Introduction

U.S. Food and Drug Administration (FDA) has proposed that process validation involves a set of documented evidence which offers paramount level of confidence in consistently producing a pharmaceutical product, such as dosage forms, meeting predetermined specifications and quality characteristics [1]. Quality standards can serve as the role of reliable codified mechanisms for managing and controlling process development. Exemplary benchmarks for quality assurance, process control, production scheduling, changeovers, maintenance, and other production activities define clear constraints on feasibility of different process technologies within the dynamic realm of real-world production settings [2]. Standardization of manufacturing procedure is valuable for ensuring process concurrency, adaptability, and formality. It provides an all-encompassing framework for holistic design and consistent development of a series of products, which ultimately has a positive impact on product quality. However, it concurrently also imposes external constraints on range of viable solutions [3]. The lacunae associated with traditional medicines is limited availability of sources and data on standardization, scientific validation, and biological studies [4].

*Ayurveda* describes numerous dosage forms through which medicament can be administered to patient. Due to its wider range of applicability (both internal and external) *Sneha Kalpana* is among most used preparations in *Ayurvedic* practices since ages. The processing of *Sneha* with *Kalka* (Coarse Powder) and *Drava Dravya* (Liquid media) for a specified time duration, until appearance of chief desired characteristics, contributes to *Sneha Kalpana.* [5] *Sneha Sidha* (fat-soluble) drugs exhibit better pharmacokinetic action compared to other dosage forms due to lipoid nature of bio-membranes, as lipid-soluble substances readily permeate into the cell membrane [6].

Pre-treatment of *Ghrita viz*., *Murchana Samskara* (fat processing) is crucial step prior *Ghrita* preparation. *Murchita Ghrita* (MG) is prepared with incorporation of *Murchana* herbs *viz*., *Phyllanthus emblica* Linn. . (Euphorbiaceae), *Cyperus rotundus* (Cyperaceae), *Curcuma longa* Linn. (Zingiberaceae), *Terminalia chebula* Retz. (Combretaceae) and *Terminalia bellirica* (Gaertn.) Roxb. (Combretaceae) and *Citrus limon* L. (Rutaceae) in equal proportion with cow's *Ghrita* and specified amount of potable water (Table 1). Scholarly textbooks of *Ayurveda* have propounded that *Ghrita* should undergo *Murchana Samskara* prior *Sneha Paka* (Oleaginous preparations), to enhance therapeutic response of *Ghrita*, as it prevents rancidity of *Ghrita* and eliminates unpleasant odour present in *Ghrita* [7,8].

**Table 1:** Contents of *Ghrita Murchana* and *Kalyanaka Ghrita* with proportions required.

Sr. No.	Plant name	Botanical name	Useful part	Ratio	Quantity
**CONTENTS OF *GHRITA MURCHANA***					
1.	*Amalaki*	*Phyllanthus emblica* Linn.	Pericarp	1/16	250 g
2.	*Haridra*	*Curcuma longa* Linn.	Rhizome	1/16	250 g
3.	*Haritaki*	*Terminalia chebula* Retz.	Pericarp	1/16	250 g
4.	*Musta*	*Cyperus rotundus* L.	Root	1/16	250 g
5.	*Nimbu*	*Citrus limon* L.	Fruit	1/16	250 g
6.	*Vibhitaki*	*Terminalia bellirica* (Gaertn.) Roxb.	Pericarp	1/16	250 g
**7.**	*Ghrita*	Clarified butter from cow milk	*-*	1	4 Kg
**8.**	Water	R.O. water	–	4	16 L
**CONTENTS OF *KALYANAKA GHRITA***					
1.	*Amalaki*	*Phyllanthus emblica* Linn.	Pericarp	1/64	29.68 (g) x 3
2.	*Brihati*	*Solanum indicum* Linn.	Whole plant	1/64	29.68 (g) x 3
3.	*Chandana (Rakta)*	*Pterocarpus santalinus* Linn.	Heart wood	1/64	29.68 (g) x 3
4.	*Dadima*	*Punica granatum* Linn.	Pericarp	1/64	29.68 (g) x 3
5.	*Danti*	*Baliospermum monatanum* Muell.	Root	1/64	29.68 (g) x 3
6.	*Daruharidra*	*Berberis aristata* Dc.	Stem	1/64	29.68 (g) x 3
7.	*Devdaru*	*Cedrus deodara* (Roxb.) Loud.	Heart wood	1/64	29.68 (g) x 3
8.	*Ela (Brihat)*	*Amomum subulatum* Roxb.	Seed	1/64	29.68 (g) x 3
9.	*Ela (Sukshma)*	*Ellateria cardamomum* Maton.	Seed	1/64	29.68 (g) x 3
10.	*Elvaluka*	*Prunus avium* L.	Stem bark	1/64	29.68 (g) x 3
11.	*Haridra*	*Curcuma longa* Linn.	Rhizome	1/64	29.68 (g) x 3
12.	*Haritaki*	*Terminalia chebula* Retz.	Pericarp	1/64	29.68 (g) x3
13.	*Kushtha*	*Saussurea lappa* C.B. Clark	Root	1/64	29.68 (g) x 3
14.	*Malati (Jati)*	*Jasminum officinale* Linn.	Flower	1/64	29.68 (g) x 3
15.	*Manjistha*	*Rubia cordifolia* Linn.	Stem	1/64	29.68 (g) x 3
16.	*Nagakesara*	*Mesua ferrea* Linn.	Stamen	1/64	29.68 (g) x 3
17.	*Padmaka*	*Prunus cerasoides* D. Don.	Heart wood	1/64	29.68 (g) x 3
18.	*Phalini (Priyangu)*	*Callicarpa macrophylla* Vahl.	Inflorescence	1/64	29.68 (g) x 3
19.	*Prisnparni*	*Uraria picta* Desv.	Root	1/64	29.68 (g) x 3
20.	*Salparni*	*Desmodium gangeticum* Dc.	Root	1/64	29.68 (g) x 3
21.	*Sariva (Shweta)*	*Hemidesmus indicus* R. Br.	Root	1/64	29.68 (g) x 3
22.	*Sariva (Krishna)*	*Cryptolepis buchnani* Roem & Schult	Root	1/64	29.68 (g) x 3
23.	*Tagara*	*Valeriana wallichii* Dc.	Root	1/64	29.68 (g) x 3
24.	*Talisa*	*Abies webbiana* Lindle.	Leaf	1/64	29.68 (g) x 3
25.	*Utpala*	*Nymphaea stellata* Wild.	Flower	1/64	29.68 (g) x 3
26.	*Vibhitaki*	*Terminalia bellirica* (Gaertn.) Roxb.	Pericarp	1/64	29.68 (g) x 3
27.	*Vidanga*	*Embelia ribes* Burm. F.	Fruit	1/64	29.68 (g) x 3
28.	*Vishala*	*Citrullus colocynthis* Scharad.	Fruit	1/64	29.68 (g) x 3
29.	*Go-Ghrita*	Clarified butter from cow milk	–	1	1900 (L) x3
30.	Water	R.O. Water	–	4	7.600 (L) x 3

*Acharya Vaghbhata* has mentioned *Kalyanaka Ghrita* [9] (KG) for management of *Kasa* (Cough), *Apasmara* (Epilepsy), *Unmada* (Insanity), and numerous other diseases. It is a medicated polyherbal *Ghrita* (Oleaginious medicament) preparation containing extracts of twenty-eight medicinal plant as mentioned in Table 1. The imperative nature of present time calls for presentation, comprehension, and implementation of development and validation of process in *Ayurvedic* pharmaceutics and therapeutics, aiming to globalize *Ayurveda*. With this perspective in mind, an endeavor was made to develop and validate standardized manufacturing procedure for KG. In this study, three batches of KG were prepared to ensure process uniformity within each batch following classical principles. Subsequently, these batches underwent comprehensive analysis to evaluate their *Sidhi Lakshana* (classical tests), physico-chemical analysis and phytochemical evaluation.

## 2 Materials and methods

### 2.1 Materials

#### 2.1.1 Procurement and authentication of medicinal drugs

Medicinal drugs mentioned for preparation of KG *viz*., *Brihati* (*Solanum indicum* Linn.), *Krishna Sariva* (*Cryptolepis buchnani* Roem & Schult), *Prisnaparni* (*Uraria picta* Desv.) *Shalparni (Desmodium gangeticum* Dc.*), Shweta Sariva (Hemidesmus indicus* R. Br.*),* and *Vibhitaki (Terminalia bellirica* (Gaertn.) Roxb.*)* were collected from herbal garden, Government Ayurved College, Raipur, Chhattisgarh, India whereas dried medicinal drugs *viz*., *Amalaki* (*Phyllanthus emblica* Linn.), *Bhadraila* (*Amomum subulatum* Roxb.), *Dadima (Punica granatum* Linn.*), Danti* (*Baliospermum monatanum* Muell.), *Daruharidra (Berberis aristata* Dc*), Devdaru (Cedrus deodara* (Roxb.) Loud*.), Ela (Ellateria cardamomum* Maton.*), Elvaluka (Prunus avium* L.*), Haridra (Curcuma longa* Linn.*), Haritaki (Terminalia chebula* Retz.*), Kushtha (Saussurea lappa* C.B. Clark*), Manjishtha (Rubia cardifolia* Linn.*), Malatimukula (Jasminum officinale* Linn.*), Nagakesara (Mesua ferrea* Linn.*), Padmaka (Prunus cerasoides* D.Don.*), Phalini (Callicarpa macrophylla* Vahl.*), Raktachandana (Pterocarpus santalinus* Linn.F.*), Tagara (Valeriana wallichii* Dc.*), Talisapatra (Abies webbiana* Lindle.*), Utpala (Nymphae astellata* Wild.*), Vidanga (Embelia ribes* Burm. F.*),* and *Vishala (Citrullus colocynthis* Scharad.*)* were procured from local market of Raipur, Chhattisgarh (Table 1). Thereafter, medicinal drugs were authenticated prior manufacturing of KG as mentioned in scholarly textbook of Dravyaguna [10]. Authentic *Ghrita* for preparation of *KG*, was sourced from local vendor associated with Amul Cow's *Ghrita* brand, manufactured by Valsad District Co-operative Milk Producers Union Limited, Vasundhara Dairy, Alipur.

### 2.2 Method

Pharmaceutical preparation of KG was carried out in accordance with principles of *Ashtanga Hrudya* [9] and Ayurvedic Formulary of India [11] in laboratory of Rasa Shastra and Bhaishajya Kalpana, Shri Narayan Prasad Awasthi Government Ayurved College, Raipur, Chhattisgarh, India.

#### 2.2.1 Pharmaceutical preparation of KG [12]

Whole pharmaceutical preparation of KG was subdivided into two sections *viz*., *Ghrita Murchana* and pharmaceutical preparation of KG. Three batches of KG were prepared by adopting *Sneha Kalpana* principles provided by *Acharya Sharangdhara* [12]. Medicinal drugs used for *Ghrita Murchana* and KG preparation with their botanical identities, parts used and specified proportions are enlisted in Table 1 respectively and specifications of equipment required for preparation of KG are mentioned in supplementary file Table 1.

##### 2.2.1.1 *Ghrita Murchana* [7]

Process of *Murchana* was followed adhering to *Ayurvedic* principles, involves pre-treatment of Cow's *Ghrita* prior pharmaceutical preparation of KG. As outlined in Table 1 specified quantity of medicinal drugs, water, and cow's *Ghrita* were utilized. Initially, *Ghrita* was poured in non-reactive stainless-steel vessel and heated over *Mandagni* (mild heat) until it reached liquid state devoid from froth and moisture. Following a slight cooling period, bolus of prescribed amount of coarsely powdered *Kalka Dravya* were added, later to which water (four times the quantity of *Ghrita*) was also poured into molten *Ghrita*. This mixture was then treated with intermittent heating until complete evaporation of water, and appearance of *Sneha Sidhi Lakshanas* (completion test). Heating duration for *Sneha Paka* was adjusted to be completed within three-day timeframe (5 h, 7 h, and 3.5 h for each day, respectively). Once, moisture-free state of *Kalka Dravya* was achieved and passed all characteristic features then, *Ghrita* was filtered through muslin cloth and subsequently *Murchita Ghrita* was stored in closed container until next used for KG preparation.

##### 2.2.1.2 Pharmaceutical preparation of *Kalyanaka Ghrita* (supplementary figure 1)

The whole process followed for preparing the Kalyanaka Ghrita is elaborated in the flowsheet given in supplementary figure 1.

###### 2.2.1.2.1 *Danti Shodhana* [13] (Purification of *Danti*)

*Danti* (*Baliospermum montanum* Muell.) is among the twenty-eight ingredients of KG. Additionally, it is also listed in Schedule E(1) of Drugs and Cosmetics Act, 1940, as a poisonous substance. Therefore, *Shodhana* (Purification) of *Danti* is necessary before adding it as *Kalka Dravya* of KG. For purification, initially, fine powder of *Pippali* (*Piper longum* Linn.) and honey were taken in equal quantity (250 g each) to form a paste. Later, 250 g of *Danti* roots were smeared with obtained paste. This mix was then covered with *Kusha* (*Desmostachya bipinnata* (L.) Stapf) and, subjected to *Swedana* (sudation) for 3-h duration at temperature of around 85 °C. Purified roots thus obtained after *Swedana* were washed and sundried. And, further used as *Kalka Dravya* for KG preparation.

###### 2.2.1.2.2 Preparation of *Kalka* [14,15]

Initially, all medicinal drugs enlisted in Table 1 were inspected for any foreign impurities manually. Subsequently, drugs were washed and sun-dried separately before subjecting to pulverize. The obtained coarse powdered drugs were then filtered using sieve shaker for particle size uniformity (Sieve No. 10/44). Coarse powder of twenty-eight medicinal drugs were then mixed and soaked in desired quantity of water to prepare a homogenous mixture. This obtained *Kalka* is then molded into a ball-shaped structure and further utilized as *Kalka Dravya* of KG.

###### 2.2.1.2.3 Preparation of *KG*

**Fig. 1:**
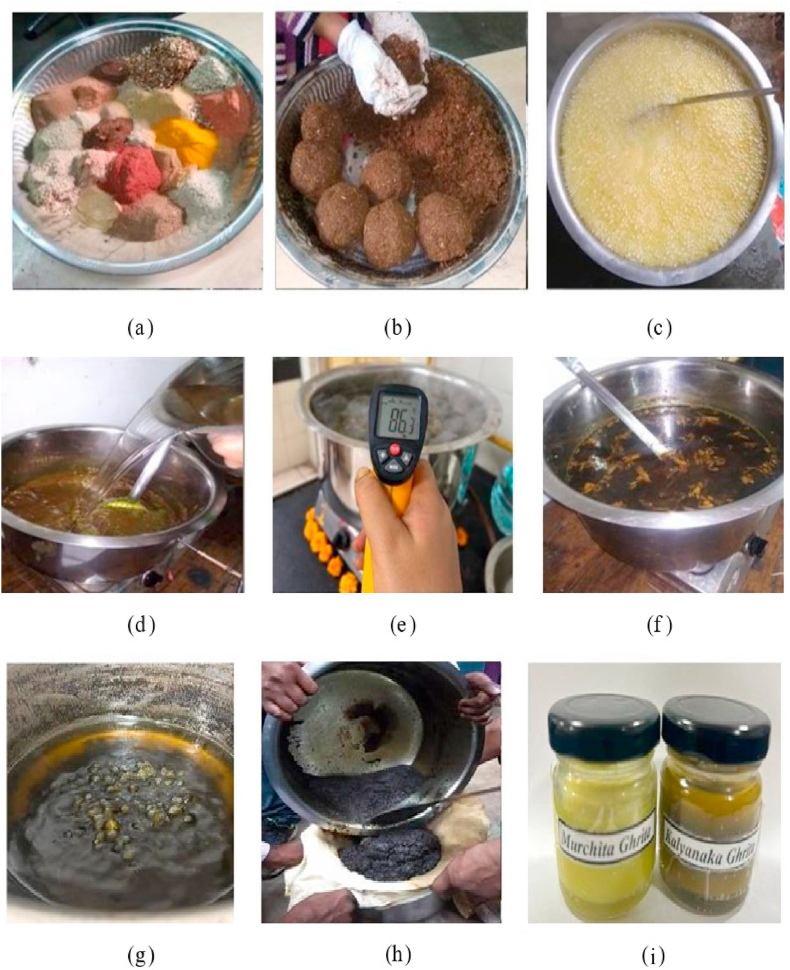
Steps of *Kalyanaka Ghrita*preparation (a) *Kalka Churna* (b) *Kalka* (c) Heating of *Murchita Ghrita* (d) Water addition (e) Temperature measuring (f) *Paka* of KG (g) *Mridu Paka* of KG (h) Filtration of KG (i) Storage of MG and KG.

Prescribed quantities of KG contents were calculated as per the standards of Ayurvedic Pharmacopoeia of India (API)/Ayurvedic Formulary of India (AFI) [16]. Firstly, *Murchita Ghrita* (MG) was emptied in stainless-steel vessel and left to liquify over *Mandagni* (mild flame) on gas stove. Thereafter, vessel containing MG was lifted from gas stove and ball-like homogeneous mixture of *Kalka Dravya* was added to it while continuously stirring. Prescribed amount of potable water (four times of MG) was also added after addition of *Kalka Dravya.* This mixture was then resubjected to *Mandagni* (intermittent mild heating) consecutively for three days, with durations of 6 h, 8.25 h, and 6.20 h on each day, respectively. Intermittent heat was provided until appearance of *Sneha Sidhi Lakshanas* (Completion Test). After achieving completion stage (Completion Test)*, Kalyanaka Ghrita* was strained through muslin cloth while in warm condition and stored in amber colored glass bottle after cooling (Fig. 1).

###### 2.2.1.2.4 *Sneha Sidhi Lakshanas* (completion test) of KG [17,18]

Consistency of *Kalka* is soft and non-sticky, making it easy to form *Varti* (wick) like shape. *Ghrita* and *Varti* (Wick) of *Kalka* when subjected to fire did not produce any crackling sound. Visible impression of finger furrows on *Varti* when pressed as shown in supplementary figure 2.

### 2.3 Analytical evaluation of KG

KG samples were analyzed for organoleptic (color, odour, taste, texture, and touch), physicochemical (acid value, iodine value, saponification value, peroxide value, refractive index, specific gravity) parameters. KG also underwent for chromatographic screening and fingerprinting through GCMS and HPLC respectively.

#### 2.3.1 Physicochemical characterization of KG

Coarse powder of medicinal drugs was used for evaluation of loss on drying while remaining parameters like pH, acid value, iodine value, saponification value, peroxide value, refractive index, and specific gravity analysis of KG was done. Qualitative and quantitative physicochemical characterization was done by adopting standard procedures described in Ayurvedic Pharmacopoeia of India [16].

#### 2.3.2 Chromatographic screening of KG

Preliminary phytochemical screening of KG was carried out for occurrence of phytochemicals such as flavonoids, alkaloids, saponins, terpenoids, phenolics and proteins by Gas Chromatography-Mass Spectrometry (GC-MS).

**GC-MS analysis conditions:** KG was subjected to Gas chromatography-Mass Spectrometry (GC-MS/MS) instrument (Plate 3) by attempting following conditions: Column, 30-m DB-WAX capillary column (0.25 mm i.d., film thickness 0.25 μm; Agilent Technologies, USA). For GC-MS detection, electron ionization system with ionization energy of 70eV was used. Helium gas (99.99%) was used as the carrier gas at constant flow rate 1.0 ml/min with a split ratio of 10:1. The oven temperature was operated according to following oven temperature: 40 °C held for 1 min, raising at rate of 20 °C per min up to 150 °C then, raising at rate of 3 °C per min, hold for 0 min and raising at rate of 20 °C per min up to 300 °C with 15 min held, injector temperature and volume 250 °C and 2 μL, respectively. The total GC running time was about 36 min. The MS operating conditions were ionization voltage 70 eV, source temperature of 250 °C, inlet line temperature 280 °C, mass scan (*m/z*)-30-500. Mass spectra of compounds were identified by comparing mass spectra obtained from their related chromatographic peaks with National Institute of Standards and Technology (NIST) mass spectral libraries.

#### 2.3.3 Chromatographic fingerprinting of KG

The standardization of KG focused on two primary plant components: curcumin, and chebulagic acid. These constituents were chosen due to their known effectiveness, which includes AChE inhibition, neuroprotective properties, anti-inflammatory effects, and antioxidant activity. The quantification of these components in KG was conducted using the HPLC method.

**HPLC conditions:** HPLC analysis was conducted using a HITACHI Chromaster system featuring a 5110-pump, a 5310-column oven, a 5430-diode array detector, and a 5210-auto sampler. The separation process utilized a HITACHI LaChrom C18 column (250 × 4.6 mm, 5 μm) at a temperature of 25 °C. The flow rate of the mobile phase was maintained at 1 mL/min, with an injection volume of 10 μL. Quantitative measurements were performed at a wavelength of 270 nm. Curcumin and chebulagic acid were identified by comparing their retention times and absorption spectra with those of standard compounds. The HPLC analysis conditions were adjusted based on previously published methodologies [19]. The mobile phase consisted of 1% acetic acid in water (A, pH 2.65) and methanol (B). The gradient elution program commenced with 90% solvent A and 10% solvent B, transitioning linearly to 50% solvent A and 50% solvent B over 25 min, followed by a 25-min washing period.

##### 2.3.3.1 Simultaneous quantification of markers

Quantification was performed by injecting 20 μL of sample solutions. Peak areas were recorded, and the concentrations of all phytoconstituents were determined using a calibration curve. The experiment was replicated three times for accuracy.

##### 2.3.3.2 Standard stock solution

10 mg of the marker compound was transferred to a standard volumetric flask and dissolved in a small quantity of methanol. It was then diluted up to 10 ml with methanol, resulting in a concentration of 1000 μg/ml. The calibration curve was generated by preparing dilutions of the standard stock solution in 10 ml volumetric flasks.

## 3 Results

### 3.1 Pharmaceutical evaluation of KG

Initially, 6.912 kg of *Ghrita* was used for *Murchana* process, resulting in production of 6.18 kg of MG. This obtained MG was then further utilized for preparation of KG in three batches. Each batch received 1.9 kg of MG, resulting in yields of 1.59 kg, 1.625 kg, and 1.54 kg of KG for respective batch (Table 2). Yield of *Danti* after *Shodhana* was 92%. Average yield of KG was calculated to be 83.41%. On average, KG preparation process was completed in 20.35 h over three days’ time-period (Table 3). Average temperature during KG preparation was 83.5 °C, with temperature ranging from 66 °C to 101 °C observed throughout the process (Table 4). The obtained KG was pale yellowish-brown in color, possessing pleasant taste, characteristic smell, with softer texture. Finished product was ascertained by disappearance of froth (*Phenashanti*), rolling the decoction residue between finger to form a wick-like structure (*Varti Pariksha*). No crackling sound was heard when this wick was subjected on fire directly (*Shabdahinata*). These tests of wick confirmed evaporation of moisture completely from *Ghrita*.

**Table 2:** Batchwise yield/loss during pharmaceutical preparation of *Kalyanaka Ghrita*

Sr. No.	Yield/loss of *Kalyanaka Ghrita*	KG batch			Average
		A	B	C	
1.	Initial quantity *of Murchita Go-Ghrita* (g)	1900	1900	1900	1900
2.	*KalyanakaGhrita* yield (g)	1590	1625	1540	1585
3.	*Kalyanaka Ghrita* yield (%)	83.68%	85.52%	81.05%	83.41%
4.	*MurchitaGo*-*Ghrita* loss (g)	310	275	360	315
5.	*MurchitaGo*-*Ghrita* loss (%)	16.31%	14.47%	18.94%	16.57%
6.	Initial weight of *Kalka* (g)	831.04	831.04	831.04	831.04
7.	Final weight of *Kalka* (g)	2050	2180	2270	2166

**Table 3:** Batchwise time duration at each stage during pharmaceutical preparation of *Kalyanaka Ghrita*.

Sr. No.	Time duration	KG batch			Average
		A	B	C	
**1.**	Moisture free stage (min)	15	18	16	16.33
**2.**	Time when *Kalka* was added (min)	20	23	21	21.33
**3.**	Time when water was added (min)	24	27	25	25.33
**4.**	*Phenshanti* (hrs)	19.30	18.30	19.45	19.01
**5.**	*Mridu Paka* stage (hrs)	20.40	19.0	20.25	19.88
**6.**	*Madhyama Paka* stage (hrs)	21.30	19.30	20.45	20.35
**7.**	*Ghrita Paka* (hrs)	21.30	19.30	20.45	20.35
**8.**	*Ghrita Paka* (days)	3	3	3	3

**Table 4:** Batchwise temperature observations at each stage of pharmaceutical preparation of *Kalyanaka Ghrita*.

Observations	KG batch (temperature in ^o^C)			
	A	B	C	Average
Loss of moisture^a^	130	140	135	135
Adding of *Kalka*	60	60	55	58.33
Boiling stage	96	98	96	96.66
*Phenshanti* stage	97	96	98	97
*Mridu Paka* stage	98	98	96	97.33
*Madhyama Paka* stage	102	101	100	101
Filtration time	82	85	80	82.33
Range of temperature	65–102	68–101	65–100	66–101
Average temperature for *Kalyanaka Ghrita* preparation	83.5	84.5	82.5	83.5

a Loss of moisture indicates moisture free stage of plain *Ghrita* which considered as achieved temperature and time duration (Table 3) to add *Kalka Dravya* for taken batch.

### 3.2 Organoleptic evaluation of KG

KG samples were observed through sensory examination for organoleptic characters such as taste, touch, colour, texture, and odour. KG was observed freshly prepared in warm conditions for best evaluation. KG exhibited softer touch, greasy texture, pleasant taste, characteristic odour with pale olive colour.

### 3.3 Physicochemical evaluation of KG

Physicochemical parameters analysis comprising loss on drying, refractive index, specific gravity, peroxide value, saponification value, iodine value, acid value, viscosity, mineral oil test and rancidity test of KG are shown in Table 5 [20,21].

**Table 5:** Batchwise physicochemical evaluation of *Kalyanaka Ghrita*.

Sr. No.	Physicochemical parameter	KG Batch		
		A	B	C
1.	Loss on drying (%)	0.1	0.1	0.1
2.	Refractive index at 40 °C	1.4542	1.4544	1.4542
3.	Specific gravity at 40 °C	0.914	0.914	0.915
4.	Peroxide value	0.4	0.4	0.4
5.	Saponification value	247.946	249.645	248.975
6.	Iodine value	33.75	32.486	33.247
7.	Acid value	3.605	3.81	3.253
8.	Viscosity	50.678	50.335	50.931
9.	Rancidity test	Absent	Absent	Absent
10.	Mineral oil	Absent	Absent	Absent

Three batches of KG showed nearly similar values for all physicochemical parameters. Loss on drying was 0.1 indicating no adulteration in raw material. Refractive index calculated for KG was 1.454. Specific gravity of KG was nearly 0.914. Peroxide value of KG samples was 0.4. Average values of saponification value, iodine value, acid value and viscosity value for three batches of KG were calculated to be 248.85, 33.61, 3.556 and 50.68 respectively. Present study revealed lower refractive index (1.454) and specific gravity (0.914) of KG whereas very minor changes were recorded in viscosity, saponification value, iodine value, and acid value in three batches of KG.

### 3.4 Chromatographic screening of KG

**Fig. 2:**
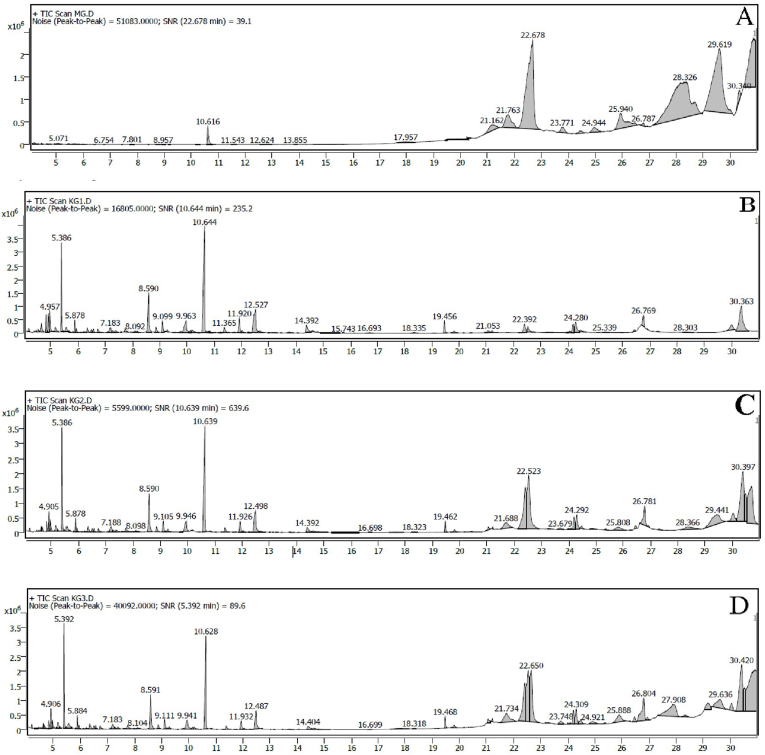
GC-MS analysis of *Murchita Ghrita* and *Kalyanaka Ghrita* (KG) formulations where, A- *Murchita Ghrita*, B- *Kalyanaka Ghrita* Batch A, C- *Kalyanaka Ghrita* Batch B and D- *Kalyanaka Ghrita* Batch C.

Significant difference was found in characteristic peak for MG and three batches of KG as shown in Fig. 2. Different compounds were identified in KG along with their retention time and area percent composition. Total 36 peaks were observed in MG out of which 20 major peaks were identified. Each batch of KG exhibited 99 peaks out of which 26 major peaks were identified in each sample. The isolated volatile compounds identified in MG were also present in KG samples along with addition of newer compounds. The compounds in all the batches of KG were same and identified as cyclopentasiloxane, phosphoric acid, parazabol, hexadecanoic acid, isopropyl myristate, dimethylthiane, butyne, carotol, muurola and pentylthiane for MG whereas, compounds identified in KG samples were citral, cyclohexene, turmerone, curlone, tetradecanoic acid, Isopropyl myristate, alantolactone, hexadecenoic acid, oleic acid, heptamethylnonane, tributyl phosphate, carboxylic acid, pyrimidine, cyclopropane, among others.

### 3.5 Chromatographic fingerprinting of KG

The results of determination of chebulagic acid content in three batches of KG averaged 17.07 mg/100 gm. The content of curcumin detected from KG solution averaged 21.49 mg/100 gm (Table 6, supplementary figure 3 and 4).

**Table 6:** Quantity of biomarkers content in *Kalyanaka Ghrita.*

Sr. No.	*Kalyanaka Ghrita* batch	Chebulagic acid/100 g	Curcumin/100 g
1.	A	18.67 mg	21.28 mg
2.	B	15.73 mg	21.42 mg
3.	C	16.81 mg	21.79 mg

## 4 Discussion

Pharmaceutical standardization is the process in which three batches of pharmaceutical product are prepared by following same process each time keeping similar amount of ingredient, instrument, temperature range, heating time and duration. Pharmaceutical development aims to meticulously design both product and its manufacturing process to consistently achieve the desired product. Insights gained from pharmaceutical development studies and manufacturing expertise offers scientific foundation for establishing specifications, and manufacturing controls [22]. Through implementation of standardization, product-to-process development can be systematically divided into well-defined, discrete, measurable, and controllable steps. This approach enables proactive identification of potential issues and allows for effective resource allocation, promoting efficient problem-solving and expedited responses to unforeseen challenges as they arise [23].

*Sneha Kalpana* (Oleaginous medicaments), represent superior class of dosage forms due to their numerous advantages like increased absorption, enhanced bioavailability, longer shelf life, and effective extraction of both fat and water-soluble active principles from medicinal plants/media within a single formulation. The primary objective of *Sneha Kalpana* is to extract lipid-soluble active principles from medicinal drugs to achieve desired therapeutic action. Additionally, these formulations aim to enhance palatability, making them easily applicable in external/internal therapeutic settings. Medicated *Ghrita* formulations are designed to encompass water-soluble, fat-soluble, and media-soluble bio-constituents, offering a comprehensive approach to deliver therapeutic benefits of various constituents in single formulation [24].

*Ghrita* is advocated as best source of nutrients among all lipids, and, *Ghrita* preparations are considered supreme as it has *Samskaranuvartana* property (Synergistic property of any substance where by addition of other substances it retains its original properties, additionally, incorporating qualities of added substances) [25].

Prior importance is mandatory to develop standard manufacturing procedures and dosage form to plan systematically*. Sneha Murchana* is pretreatment procedure of *Sneha* before final preparation in which *Sneha* is boiled with coarse powder of advised medicinal drugs and appropriate amount of water to eliminate *Ama* and *Gandha Dosha* of *Ghrita*, as indicated in Table 1. *Ghrita Murchana* is recommended in *Ayurveda* for internal medications to enhance therapeutic efficacy of *Ghrita* formulations. Herbs used for *Murchana Samskara* possess antioxidant and anti-lipid peroxidation properties, thus, preventing primary oxidation of *Ghrita* [26]. *Murchana* process of *Ghrita* not only maintain ratio of unsaturated and saturated fats but also modifies the solubility pattern and absorbability of *Ghrita* formulation [27].

Previous studies have shown positive impact of *Murchana* process on enhancing the therapeutic efficacy, acceptability, stability, and overall shelf life of *Ghrita* preparations [26,28].

*Drava Dravya* (Liquid media) play an important role in *Sneha Paka* as they enhance transfer of active phytoconstituents from medicinal drugs (*Kalka Dravya*) and subsequently, active phytoconstituents are transferred to *Sneha*. This transfer of active phytoconstituents is well explained by mass transfer theory. Soluble active phytoconstituents are transferred from solid substance (*Kalka Dravvas*) to liquid media (*Drava Dravya*). Transport of active phytoconstituents occurs in two phases. Initially, active phytoconstituents move through liquid's boundary via molecular diffusion. Once these active phytoconstituents have crossed boundary layer, then, mass transfer will take place by bulk movement of liquid, known as Eddy diffusion. In second phase, active phytoconstituents are transported from liquid media (*Drava Dravya*) to fluid (*Sneha*) through fluid/fluid mass transfer. This theory is applicable for scenarios where mass transfer takes place between two immiscible fluids. In such cases, there exist boundary layers of both fluids on each side of interface, where, the concentration gradients depend upon on diffusion coefficients of two medias [29]. In the present study, Amul cow *Ghrita* was taken as baseline standard due to its universal availability and therapeutic value as compared to other available sources of *Ghrita* in the market [30].

Initially, *Ghrita Murchana* was carried out wherein Cow's *Ghrita* was boiled with coarse powder of medicinal drugs with desired quantity of water. Process of *Murchana* enhances the absorbability of *Ghrita*. Hence, *Murchita Ghrita* obtained after *Murchana Sanskara* possess more medicinal properties as compared to without *Murchana Sanskara*. *Murchana Sanskar* refines Cow's *Ghrita*, by removing undissolved solids, moisture content/factor causing rancidity (*Ama Dosha*), undesirable odour (*Gandha Dosha*), free fatty acids, phosphatides etc which alters physico-chemical properties of Cow's *Ghrita*. This process in turn potentiates Cow's *Ghrita* by inducing antioxidant properties, inhibiting lipid-peroxidation, making it easily digestible and additionally, drug absorbability, assimilation and shelf life are remarkably enhanced.

During pharmaceutical preparation process, it was also observed that utilization of coarse powder of medicinal drugs instead of fine powders for preparation of *Kalka* in *Ghrita Paka* resulted in higher yield. During *Sneha Sidhi Lakshana* (Completion Test) stage, it was observed that *Varti* (Wick) made from coarse powder medicinal drugs was easier to form. *Kalka* exhibited quicker miscibility and imparted its colour to Cow's *Ghrita* when added by making ball shaped paste with addition of r. o. water. So, instead of using medicinal drugs in dry form they were used in form of *Kalka* (paste) for *Sneha Paka*.

The average temperature on completion of *Murchana* was 83.3 °C and loss in Cow's *Ghrita* after *Murchana Sanskara* was 8.8%. Average time taken for *Ghrita Murchana* was 16.63 h which was completed in three days timespan, by following intermittent heating pattern. In this study, loss of *Ghrita* was observed more, due to four reasons (i) dry form of drugs used; (ii) particle size of *Kalka Dravya* resulting in enhanced surface area for more absorption of Cow's *Ghrita* in *Kalka Dravya*, (iii) nature of certain *Kalka Dravya* like *Triphala* is sticky which increase the loss of *Sneha* during filtration and (iv) partial loss may be due to absorption by cloth used for filtration.

Pharmaceutical processing of *KG* was done following the general principle of *Sneha Paka Kalpana*. At first *Danti Shodhana* was conducted since, it is mentioned in Schedule E1 drugs of Drugs and Cosmetics Rules, 1945. *Ghrita Paka* was completed within three days as per classical principle since water was used as liquid media used for KG preparation. Since, KG was prepared for internal administration, intermittent heating was carried out up to *Madhyama Paka* stage only. Probably, during this stage transfer of phytoconstituents would be at an optimum level. Overheating up to *Khara Paka* stage is not recommended for internal administration to avoid burning of *Kalka Dravya* and degradation of active phytoconstituents as it may produce burning sensation in stomach. In context of *Ghrita Paka*, scholarly textbooks of *Ayurveda* have advocated not to go beyond *Madhyama Paka* except for *Abhyangam* (massage) purpose, presumably to retain active phytoconstituents level in *Sneha* and make the *Ghrita* readily absorbable through body channels. Therefore, *Madhyama Paka* heated *KG* (approx. heating range 83.3 °C) is recommended for internal application in *Unmada*, *Apasmara* and other diseases. *Ghrita Sidhi Lakshana* observed during *Ghrita Murchana* and *KG* preparations are shown in supplementary figure 2. Water was specified as *Drava Dravya* for preparation of MG and KG. *Sneha Paka* principles specified by *Acharya Sharangdhara* suggests three days intermittent heating period when water is taken as *Drava Dravya*. Hence, average duration for preparation of *Murchita Ghrita* and *Kalyanaka Ghrita* was 16.46 h and 20.45 h divided into three days timespan respectively. Average temperature range on completion of *Sneha Paka* for *Kalyanaka Ghrita* was 83.3 °C. Average loss of *Ghrita* in final product was 8.3 % in KG. Due to transfer of various phytoconstituents of medicinal drugs and other pigments the colour of KG was pale yellowish brown having pleasant taste, characteristic smell and softer on touch.

Palatability of product is found to be dependent upon sensory characteristics. The sensory analysis is pilot basis for quality control as well as quality assurance of *Ghrita* based products in general [31]. KG retained characteristics of *Ghrita* used for preparation i.e., oily consistency and granular appearance. However, it was observed that palatability of *Ghrita* was increased after *Murchana* process but reduced after KG preparation. *Murchana* process altered organoleptic properties (colour, odour, and taste) of *Ghrita* resulting in complete disappearance of characteristic odour, granular and oily consistency of *Ghrita* and made KG formulation homogeneous and smooth.

Prepared three batches of KG formulations were evaluated for physicochemical properties for oleaginous preparations such as viscosity, loss on drying, refractive index, specific gravity, peroxide value, saponification value, iodine value, rancidity test, mineral oil, and acid value. Ratio of weight of material to weight of water for constant volume is specific gravity. Weight of lipid material is affected by basic constitution, dissolved constituents used during the processing of formulation. The less liquid content in formulation increases the life span and thus its therapeutic efficacy. It also changes because of temperature during processing [32,33]. No significant changes in specific gravity were observed among three batches of KG (Table 5).

Viscosity affects the appearance and consistency of any sample as it is a measure of resistance of flow of *Ghrita* formulations when an external force is applied. Viscosity of KG was found to be 50.648 on an average for three batches. Occurrence of low foreign matter in plant ingredients indicates less adulteration of raw materials. Lower values of loss on drying indicates lower moisture content indicating minimum enzymatic and microbial activity with extended shelf life. Values for loss on drying are 0.1 in three batches of KG indicating the purity of raw material used. Refractive index represents behavior of light in any medium, which is used to determine concentration of solutes in an aqueous solution. Refractive index is inversely proportional to chain length where presence of double bond elevates refractive index [34].

The initial evidences of rancidity in unsaturated fats and oils are provided by peroxide value, an index of the degree of auto-oxidation [35], helps to assess spoilage of product [36]. The peroxide value was determined to obtain initial evidence of rancidity. The calculated peroxide value of KG was 0.4. Lower peroxide value of KG indicates minimum rancidity. Antioxidant properties of *Murchana* herbs seemed to be offering protective effect against rancidity of processed *Ghrita* [37].

Acid value is the free fatty acid (FFA) present in *Ghrita* is related to its stability. Formation of FFA might be an important factor for rancidity of *Ghrita*. FFAs are formed due to hydrolysis of triglycerides and may be promoted by the reaction of *Ghrita* with moisture [38]. Presence of fatty acid profile affects the quality, stability, and shelf life of *Ghrita*. The FFA present in *Ghrita* indicates its identity and purity. The average acid value of KG for three batches is 3.556, indicating hydrolysis of *Ghrita* during the process of *Snehapaka*. The reaction may be promoted by the triglycerides present in *Ghrita* with active phytoconstituents present in KG, resulting in formation of glycerol and free fatty acids. High amounts of free fatty acid (Acid Value) favor a decrease in quality of *Ghrita*.

The iodine value indicates quantity of iodine absorbed at unsaturation which signifies the degree of unsaturation of the *Ghrita* formulations [39]. The formulation with higher iodine value is more reactive and susceptible to the oxidation. Iodine values determine unsaturated fatty material present in *Ghrita*. Higher iodine number represents greater unsaturated bonds present in fat. Unsaturated fat supplementation increases total dietary energy intake to recommended levels, and it has no adverse impact on blood lipids. It improves nutritional status and reduces systemic inflammation [40]. Average iodine value of KG is found to be 33.161 for three batches of KG.

Saponification value gives an indication of number of fatty acids and their average molecular weight in *Ghrita* formulations. More the fatty matter content or more the carboxylic functional group per unit mass, there will be more chances of rancidity incidences, hence reduced shelf life and therapeutic value [39,41]. Longchain fatty acids found in fat have low saponification value, that is, shortchain fatty acids (SCFAs) have a high saponification value. Short chain fatty acids are readily absorbed; a greater increase in SCFA production and potentially a greater delivery of SCFAs, specifically butyrate, to the distal colon, may result in protective effect [42]. The average saponification value of KG was found to be 248.85. This increase in saponification value of KG formulation could be attributable to interactions between various *Ghrita* components and photoactive constituents.

GC–MS analysis was conducted to observe number of compounds present in final prepared KG formulation and to provide additional evidence of *Murchana* process by comparing the results with KG [43]. Upon comparing GC-MS data obtained from reported studies of cow *Ghrita* with present KG, the spectra show the presence of newer phytoconstituents in KG compared to cow *Ghrita*. This clearly reflects that processing *Sneha* with different medicinal plants enriches the final product and increases wide range of therapeutics compared to plain *Ghrita* [44]. GC-MS spectra of three batches of KG is presented in Fig. 2 and compared with reference to area per cent of major peaks. Total 99 number of compounds were detected in GC–MS for KG. KG samples clearly showed higher contents of volatile compound peak area than MG sample. The illustration of these compounds depicted a clear view about addition of newer compounds during the processing after *Ghrita Murchana*. The results of major volatile compounds observed are enlisted in supplementary file Table 2. The volatile compounds observed in three batches of KG resulted similar compounds (only major compounds have been considered) but with slightly different peak areas. Each peak represented a particular volatile compound exhibited in majority in samples. The peaks obtained were expressed in terms of retention time (in second) versus intensity (peak area, proportional to concentration of relevant compounds present in the samples).

Presence of total 36 volatile compounds were detected in MG. Out of which 20 compounds were selected based on their higher percent relative peak area and were identified as the major possible volatile markers for MG. These possible compounds were cyclopentasiloxane, phosphoric acid, parazabol, hexadecanoic acid, isopropyl myristate, dimethylthiane, butyne, carotol, murola and pentylthiane. Similarly, different batches of KG showed the presence of 99 compounds. Out of which, KG batch A showed 26 major compounds whereas batch B and C of KG showed presence of 28 major compounds. Predominance of compounds such as citral, cyclohexene, turmerone, curlone, tetradecanoic acid, Isopropyl myristate, alantolactone, hexadecenoic acid, oleic acid, heptamethylnonane, tributyl phosphate, carboxylic acid, pyrimidine, cyclopropane, among others. The presence of MG compounds was seen in KG samples with addition of newer compounds due to further processing of *Murchita Ghrita.*

The complexity of herbal formulations poses a significant obstacle to standardization due to their intricate chemical composition. Unlike synthetic medications, this complexity hinders the creation of appropriate analytical techniques for standardization purposes. Marker-based standardization has been acknowledged as a beneficial technique for standardizing polyherbal formulations [45]. Hence, in the present study two biomarkers were taken into consideration for their quantitative estimation through HPLC. Determination of chebulagic acid and curcumin revealed the presence of 0.17% chebulagic acid and 0.02% curcumin on an average, was detected in all batches of *KG*.

## 5 Conclusion

*Sneha Kalpana* holds significant importance as widely recognized and extensively utilized form of medication within *Ayurvedic* system of medicine. Process validation of manufacturing procedure served as a valuable source to ensure quality and adherence of Good Manufacturing Practices (GMPs). Present study suggests that for *Kalka* preparations, use of coarse powder is preferable over fine powders. Preparation of *Kalyanaka Ghrita* (KG) was completed within three-day's timeframe, with total duration of heat applied for 20.35 h with an average temperature of 83.5 °C for batch size of 1.9 L *Murchita Ghrita*. Yield of *KG* was approximately 83.41%, with corresponding loss of 16.57% *Murchita Ghrita*. GCMS analysis of three batches of KG shows presence of 99 peaks with similar compounds. HPLC estimation revealed average 0.17% of chebulagic acid and 0.02% curcumin in all the batches of *KG*. Findings of the present study emphasize the importance of maintaining uniformity in operative procedures, making the development and process validation of manufacturing procedure for *KG.* The values provided through the tentative quality specifications of the present study, including GC-MS, HPLC, and other physicochemical tests, can serve as a minimum baseline criterion for future works and batches prepared using the same procedure. This data could also be integrated into the Ayurvedic Pharmacopoeia of India (API) to standardize using these specifications. Additionally, the academia and pharmaceutical industry may adopt these procedures to ensure consistency and quality across each batch.

## Sources of funding

The authors are grateful to the Directorate of 10.13039/501100008802AYUSH, Raipur Chhattisgarh for their financial support in the successful conduction of this study.

## Author contributions

Yashika Singh: Contributed to the data curation, validation and writing original draft. Amzad Ali Ansari: Contributed to the visualization, availing resources and preparing first draft. Rajendra Prasad Sharma: Revised the content critically by reviewing and editing. Saroj Moreshwar Parhate: Contributed to the investigation, supervision, reviewing and editing of intellect content. Thakur Rakesh Singh: Contributed to the conceptualization, methodology, project administration, reviewing and editing of article.

## Declaration of generative AI in scientific writing

The authors declare that no AI tools were used for writing the manuscript.

## Declaration of competing interest

The authors declare the following financial interests/personal relationships which may be considered as potential competing interests:

NONE.
