# Supplementary material for: Product development and characterization of a lipid-based Ayurvedic polyherbal formulation: Kalyanaka Ghrita
Source: J Ayurveda Integr Med. 2024 Oct 15;15(5):101011. doi: 10.1016/j.jaim.2024.101011 (PMC11525137; doi:10.1016/j.jaim.2024.101011)
Supplement: Multimedia component 1 [file mmc1.doc]

**Supplementary file**

Supplementary figure 1. Schematic flowchart for pharmaceutical preparation of *Kalyanaka* *Ghrita*.


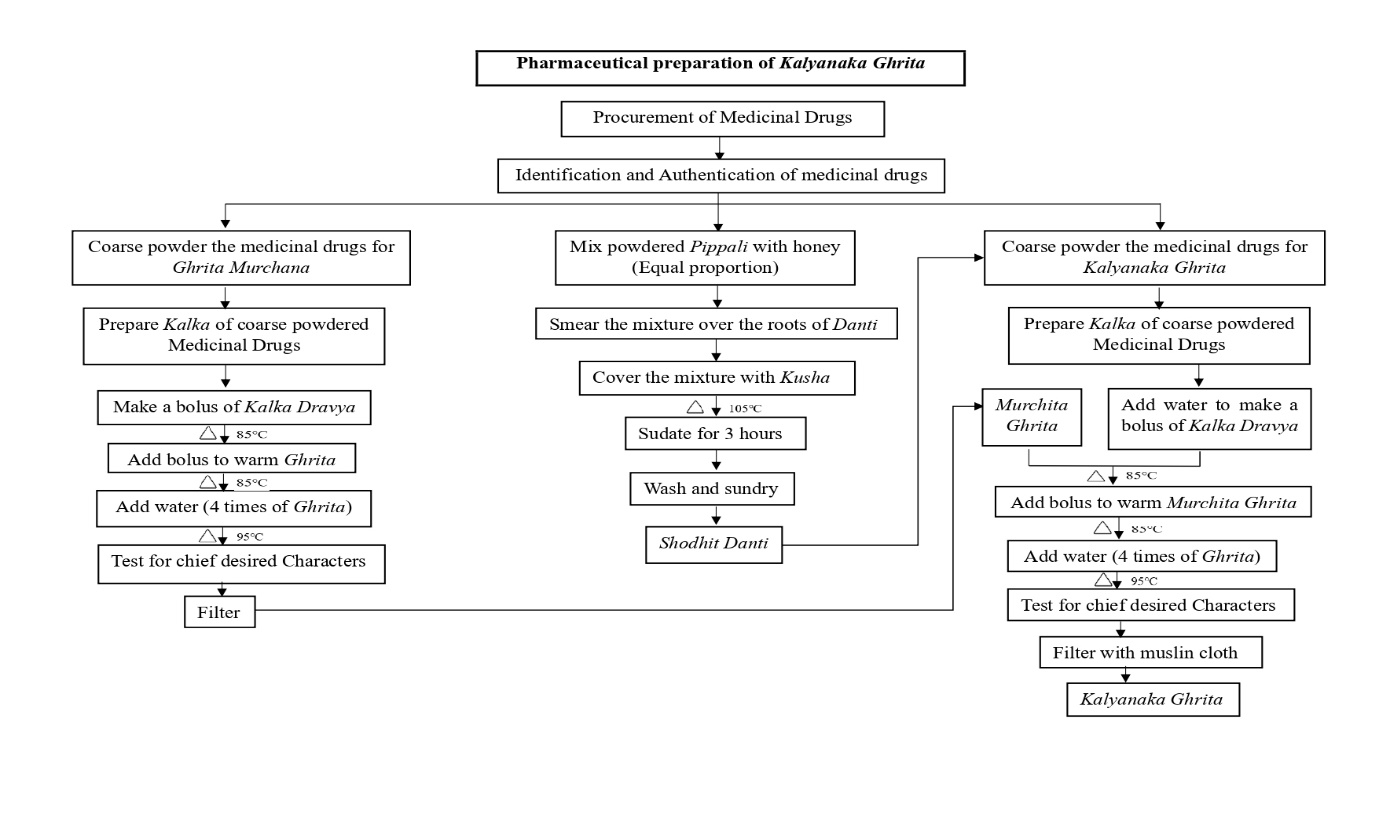


Supplementary figure 2. *Sneha Siddhi Lakshana* (Test for completion of *Sneha*)

(a.) *Madhyama Paka* (b.) *ShabdahinoAgni Nikshipta* (c.) *Varti* test

**
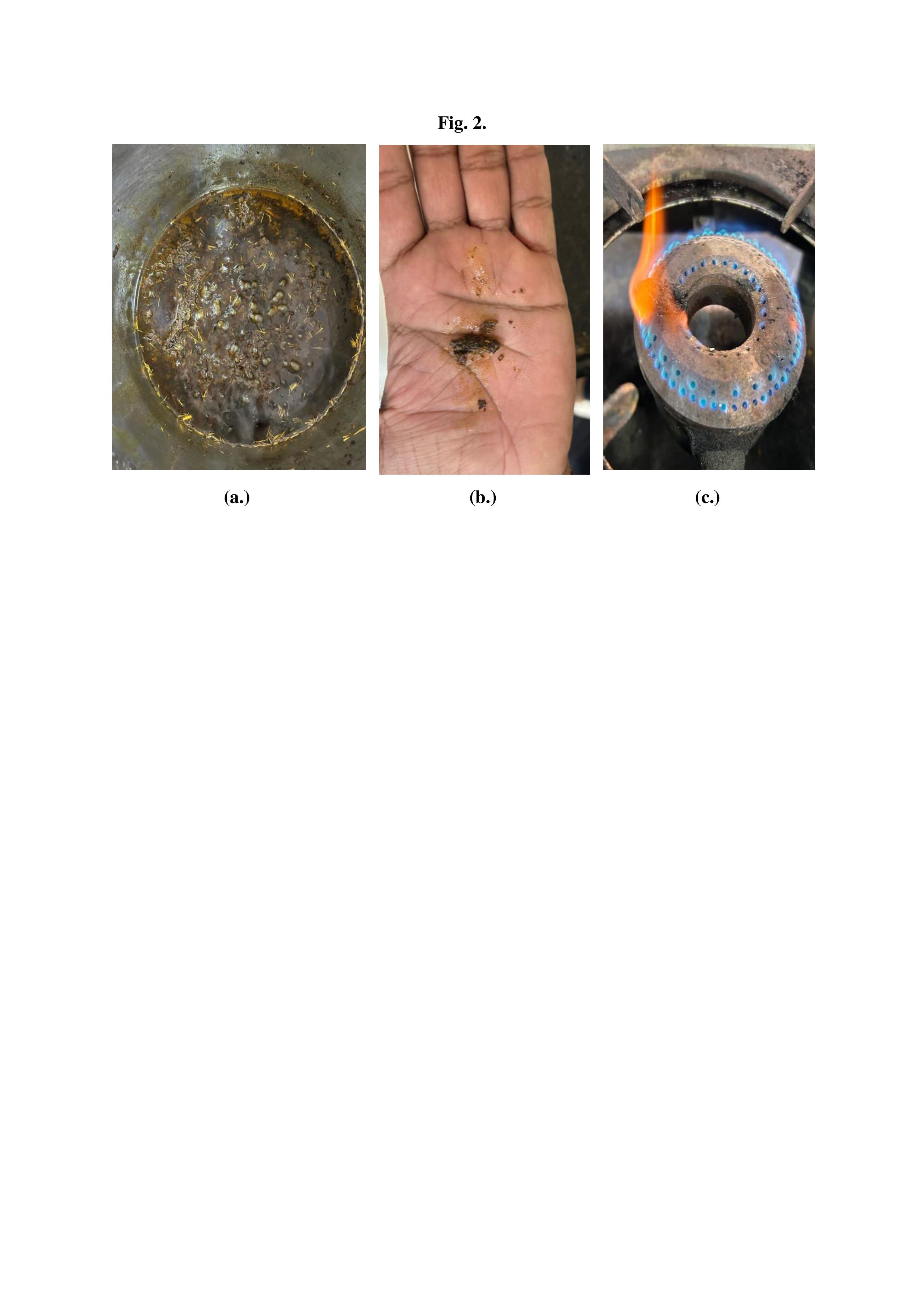
**

Supplementary figure 3. Chromatogram of *Kalyanaka Ghrita* for Curcumin (a) *Kalyanaka* *Ghrita* Batch A, (b) *Kalyanaka* *Ghrita* Batch B and (c) *Kalyanaka* *Ghrita* Batch C.

**
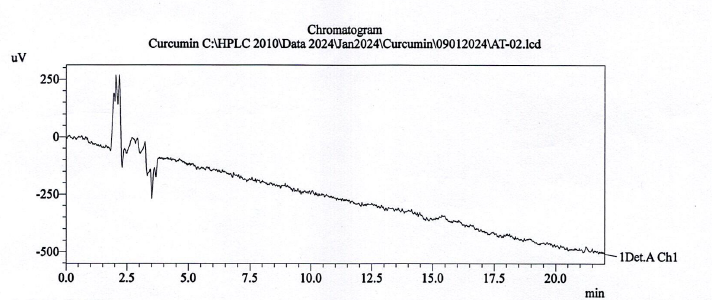
**

**(a.)**


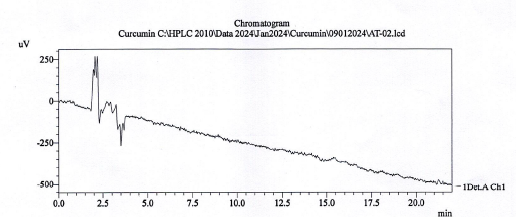


**(b.)**

**
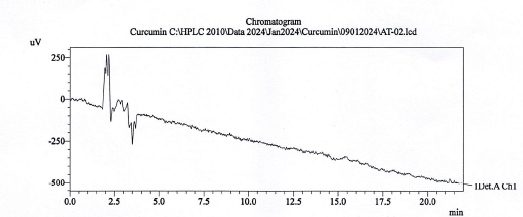
**

**(c.)**

Supplementary figure 4. Chromatogram of *Kalyanaka Ghrita* for Chebulagic acid (a) *Kalyanaka* *Ghrita* Batch A, (b) *Kalyanaka* *Ghrita* Batch B and (c) *Kalyanaka* *Ghrita* Batch C.

**
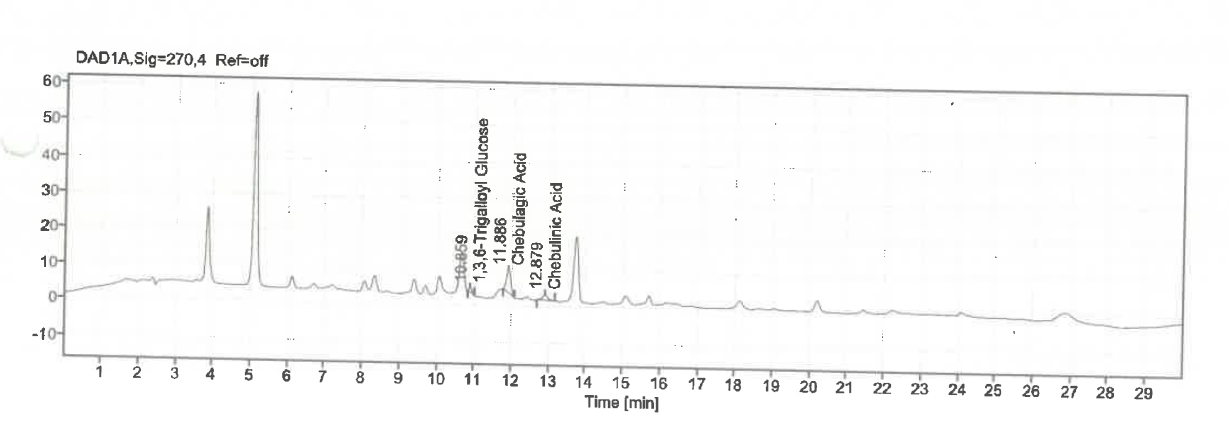
**

**(a.)**

**
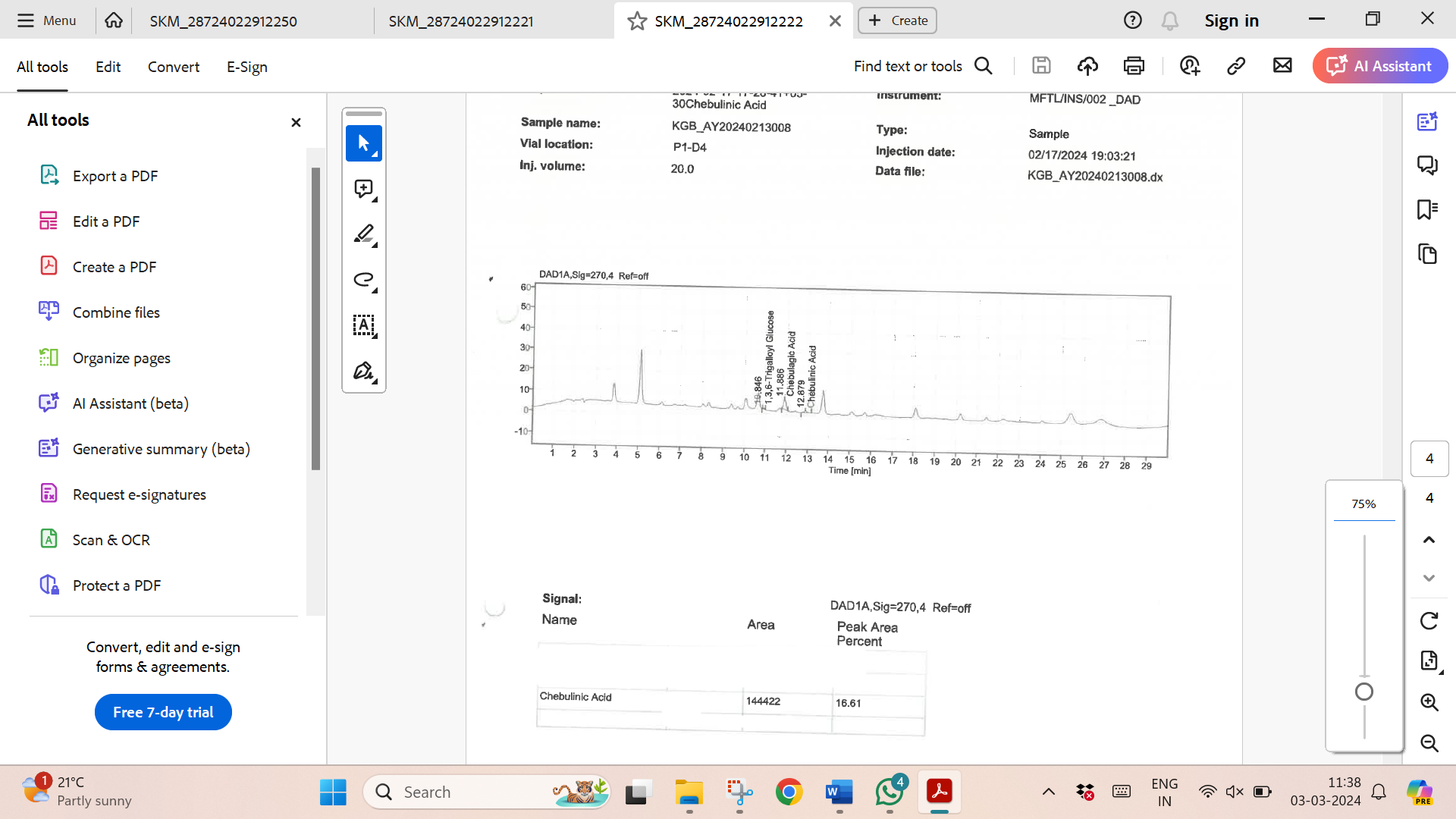
**

**(b.)**

**
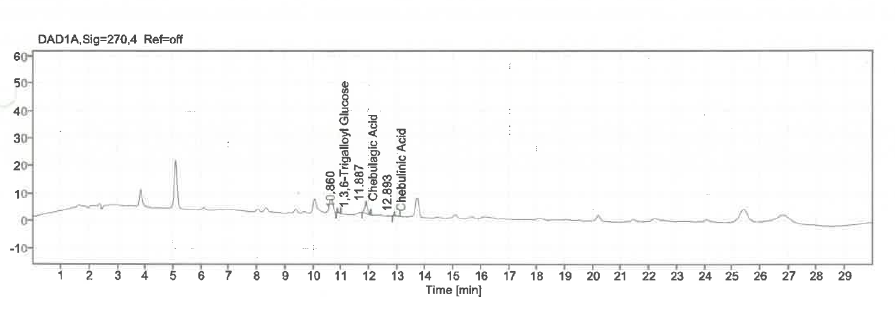
**

**(c.)**

**Supplementary table 1. Equipment specifications used for *Kalyanaka Ghrita* preparation.**

| **Sr. No.** | **Equipment** | **Nature** | **Dimension** | **Capacity** |
| --- | --- | --- | --- | --- |
| **1.** | Vessel | Stainless steel | Height-9.5 inch | 23 liters |
|  |  |  | Diameter-14.5 inch |  |
|  |  |  | Weight- 3.700 kg |  |
| **2.** | Ladle | Stainless steel | Weight- 200 gm | **-** |
|  |  |  | Length-17.5 inch |  |
| **3.** | Heating Device | Gas cylinder with stove | - | 14.2 kg |
| **4.** | Cloth | Muslin | - | 2 meters |
| **5.** | Thermometer | Electric cell operated | - | 1400℃ |
| **6.** | Measuring Jar | Plastic | - | 250 ml,  500 ml |
| **7.** | Weighing balance | Electronic balance | - | 100 kg |

**Supplementary table 2. List of compounds identified on Gas Chromatography- Mass Spectrometry (GCMS) for *Murchita Ghrita* and three batch of *Kalyanaka Ghrita.***

| **Sr. No.** | **Compound** | **% area** | | | |
| --- | --- | --- | --- | --- | --- |
|  |  | **MG** | **KG batch** | | |
|  |  |  | **A** | **B** | **C** |
|  | (E)-Atlantone | NA | 2.02 | 0.23 | 0.66 |
|  | 1,4-Benzenedicarboxylic acid, bis(2-ethylhexyl) ester | NA | 8.34 | 5.55 | 2.96 |
|  | 1,4-Methanocycloocta[d]pyridazine | 0.03 | 0.12 | 0.49 | 1.01 |
|  | 2-Pentadecanone | NA | 4.45 | 3.24 | 1.44 |
|  | 3H-Pyrazole-3-carboxylic acid | NA | 1.72 | 0.57 | 0.89 |
|  | 3-n-Hexylthiolane, S,S-dioxide | 0.01 | 0.05 | 0.12 | 0.14 |
|  | 4-n-Hexylthiane, S,S-dioxide | 0.4 | 0.88 | 1.27 | 0.18 |
|  | Alantolactone | NA | 11..43 | 7.28 | 3.02 |
|  | Aromandenderene | NA | 1.98 | 0.49 | 1.01 |
|  | Arsenous acid, tris(trimethylsilyl) ester | NA | 0.6 | 0.06 | 0.07 |
|  | aR-Turmerone | 0.03 | 31.26 | 23.89 | 11.79 |
|  | Azulene | NA | 2.07 | 2.05 | 0.94 |
|  | Bacteriochlorophyll-c-stearyl | NA | 1.72 | 3.35 | 1.52 |
|  | β-D-Manofuranoside | NA | 0.08 | 0.07 | 0.67 |
|  | Borabicyclononane | NA | 0.33 | 0.48 | 0.39 |
|  | Boric acid (H3BO3), tripentyl ester | 0.03 | 1.48 | 0.83 | 29.74 |
|  | Borinic acid | 0.01 | 0.19 | 0.57 | 0.11 |
|  | Cardinene | NA | 2.07 | 1.25 | 0.94 |
|  | Caryophyllene | 0.03 | 2.03 | 1.46 | 0.76 |
|  | Cholest-5-en-3-ol, (3.alpha.) | NA | 11.29 | 12.43 | 11.81 |
|  | Citral | 0.11 | 2.08 | 4.94 | 2.07 |
|  | Corymbolone | NA | 0.57 | 0.84 | 1.98 |
|  | Curlone | 0.01 | 7.66 | 5.71 | 2.63 |
|  | Cyclododecyne | 0.02 | 2.29 | 1.81 | 1.91 |
|  | Decanoic acid | 0.14 | 5.16 | 5.18 | 2.62 |
|  | Dicyclohexyl ethyl phosphate | NA | 0.06 | 0.07 | 0.41 |
|  | Dodecanoic acid | 5.26 | 5.76 | 5.1 | 2.41 |
|  | Guaiol | NA | 0.84 | 0.96 | 0.72 |
|  | Gurmacrene | NA | 1.73 | 1.84 | 1.92 |
|  | Hydroxyvalerenic acid | NA | 2.3 | 7.28 | 5.43 |
|  | Isopropyl myristate | 3.55 | 100 | 67.74 | 29.68 |
|  | Longifolene | NA | 4.85 | 4.23 | 2.24 |
|  | Methyl 11,12-tetradecadienoate | 0.11 | 0.22 | 0.07 | 0.06 |
|  | Methyl 2-((bis(isopentyloxy)phosphoryl)oxy)-3,3,3-trifluoropropanoate | 3.3 | 2.64 | 0.86 | 0.07 |
|  | Naphthalene | NA | 1.98 | 1.89 | 1.01 |
|  | n-Hexadecanoic acid | 0.14 | 38.12 | 23.67 | 8.6 |
|  | Octadecanoic acid | NA | 1.52 | 2.76 | 1.85 |
|  | Oleic acid | NA | 9 | 6.99 | 7.21 |
|  | Palmitoleic acid | NA | 0.88 | 1.54 | 2.34 |
|  | Pentadecanoic acid | NA | 0.83 | 0.23 | 0.12 |
|  | Phosphonic dichloride | NA | 5.15 | 0.64 | 2.3 |
|  | Phosphoric acid, dipentyl octyl ester | 1.14 | 0.46 | 0.13 | 2.87 |
|  | Phosphoric acid, diundecyl ethyl ester | NA | 1.49 | 13.97 | 5.16 |
|  | Tetradecanoic acid | 3.55 | 17.91 | 0.12 | 5.67 |
|  | Thymol | NA | 0.12 | 4.45 | 2.3 |
|  | trans-2-methyl-4-n-pentylthiane, S,S-dioxide | 0.54 | 0.24 | 0.12 | 0.02 |
|  | trans-Verbenol | 0.11 | 7.84 | 1.3 | 1.53 |
|  | Tributyl phosphate | NA | 1.3 | 0.83 | 5.16 |
|  | Tris(tert-butyldimethylsilyloxy)arsane | NA | 0.13 | 0.13 | 0.18 |
|  | Uvidin C | NA | 0.57 | 0.45 | 0.12 |
|  | γ-Muurolene | NA | 0.56 | 2.29 | 1 |
